# Embedding MSCs in Si-HPMC hydrogel decreased MSC-directed host immune response and increased the regenerative potential of macrophages

**DOI:** 10.1093/rb/rbac022

**Published:** 2022-04-25

**Authors:** Christelle Demarquay, Lara Moussa, Gildas Réthoré, Fabien Milliat, Pierre Weiss, Noëlle Mathieu

**Affiliations:** Human Health Department, IRSN, French Institute for Radiological Protection and Nuclear Safety, SERAMED, LRMed, Fontenay-aux-Roses 92262, France; Human Health Department, IRSN, French Institute for Radiological Protection and Nuclear Safety, SERAMED, LRMed, Fontenay-aux-Roses 92262, France; Faculté de Chirurgie Dentaire, Regenerative Medicine and Skeleton (RMeS) Laboratory, Université de Nantes, Nantes 44042, France; Human Health Department, IRSN, French Institute for Radiological Protection and Nuclear Safety, SERAMED, LRMed, Fontenay-aux-Roses 92262, France; Faculté de Chirurgie Dentaire, Regenerative Medicine and Skeleton (RMeS) Laboratory, Université de Nantes, Nantes 44042, France; Human Health Department, IRSN, French Institute for Radiological Protection and Nuclear Safety, SERAMED, LRMed, Fontenay-aux-Roses 92262, France

**Keywords:** Si-HPMC hydrogel, mesenchymal stromal cells, host immune response, regenerative medicine

## Abstract

Embedding mesenchymal stromal cells (MSCs) in biomaterial is a subject of increasing interest in the field of Regenerative Medicine. Speeding up the clinical use of MSCs is dependent on the use of non-syngeneic models in accordance with Good Manufacturing Practices (GMP) requirements and on costs. To this end, in this study, we analyzed the *in vivo* host immune response following local injection of silanized hydroxypropyl methylcellulose (Si-HPMC)-embedded human MSCs in a rat model developing colorectal damage induced by ionizing radiation. Plasma and lymphocytes from mesenteric lymph nodes were harvested in addition to colonic tissue. We set up tests, using flow cytometry and a live imaging system, to highlight the response to specific antibodies and measure the cytotoxicity of lymphocytes against injected MSCs. We demonstrated that Si-HPMC protects MSCs from specific antibodies production and from apoptosis by lymphocytes. We also observed that Si-HPMC does not modify innate immune response infiltrate *in vivo*, and that *in vitro* co-culture of Si-HPMC-embedded MSCs impacts macrophage inflammatory response depending on the microenvironment but, more importantly, increases the macrophage regenerative response through *Wnt*-family and *VEGF* gene expression. This study furthers our understanding of the mechanisms involved, with a view to improving the therapeutic benefits of biomaterial-assisted cell therapy by modulating the host immune response. The decrease in specific immune response against injected MSCs protected by Si-HPMC also opens up new possibilities for allogeneic clinical use.

## Graphical Abstract

**Figure:**
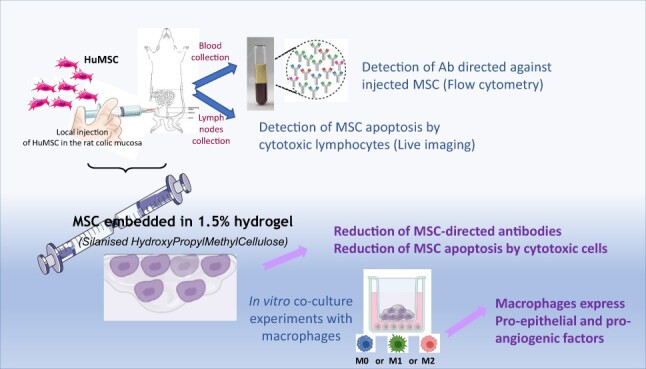

## Introduction

Cell therapy, and particularly mesenchymal stromal cell (MSC) therapy, is considered to be a promising option for treating various disorders and diseases that have hitherto remained untreated or were thought to be incurable. A plethora of reports based on pre-clinical studies demonstrates their therapeutic properties with regard to various pathologies induced in animal models, due to the secretion of a wide range of paracrine factors, collectively referred to as the secretome [1]. This broad panel of molecules induces a pleiotropic effect by acting on the surrounding environment to boost intrinsic tissue repair responses (angiogenesis, tissue-specific cell proliferation and progenitor recruitment). MSC secretome also exerts immunomodulatory properties: inhibiting activation and proliferation of T and B cells and promoting differentiation of monocytes into anti-inflammatory Type 2 macrophages, through the secretion of anti-inflammatory molecules [2, 3]. Direct action of MSCs on immune cells has also been described, focusing on monocytes [4, 5] or T cells [6]. Moreover, interaction between MSCs and the host microenvironment can modify their secretome. Indeed, several studies have demonstrated that the inflammatory microenvironment is necessary to stimulate MSCs in order to achieve their full therapeutic benefits [7, 8].

While all the action mechanisms of the therapeutic benefits of MSCs have yet to be elucidated, encouraging results obtained in pre-clinical models have given rise to hundreds of clinical trials [9, 10]. Today, 10 years on, the safety of this therapy appears to be established [11]. However, the results are more elusive when it comes to clinical efficacy [12]. Indeed, the benefits of MSC-based therapy are not stable in the long term and the final outcomes manifest high inter-patient variability. One of the causes of these therapeutic limitations is poor engraftment and survival of the transplanted cells in the damaged tissue to exert their full therapeutic benefit. Several factors can influence the retention and viability of the infused cells, including mechanical stress during the injection procedure in organs, cell apoptosis caused by anoikis (i.e. lack of cell adhesion) and endogenous environmental factors such as hypoxia, inflammatory microenvironment or host immune response [13]. To overcome these drawbacks, various approaches have been developed. The genetic modification of MSCs to overexpress pro-survival factors and MSC pre-conditioning have improved MSC engraftment and been correlated with increased therapeutic efficiency [14, 15]. With this in mind, in a previous study, MSCs were embedded in a silanized hydroxypropyl methylcellulose (Si-HPMC) hydrogel to improve retention and survival in the colon damaged by irradiation. Si-HPMC hydrogel has been designed and characterized for injectable cell delivery using the operative catheter of a colonoscope. We showed that encapsulated MSCs were viable after more than 21 days in culture and maintained their paracrine abilities. *In vivo* experiments demonstrated higher cell engraftment, associated with improvement of the colonic structure and the epithelial barrier function [16]. However, the action mechanisms were not fully understood, particularly those involving the immune system. MSCs have been described as immune-privileged cells, due to their low expression of human leukocyte antigen (HLA) major histocompatibility complex Class I, their absence of expression of costimulatory molecules and expression of HLA-G [17]. However, despite their immune-privileged status, several studies have highlighted the development of an immune response against allogeneic MSCs [13, 18]. Although few studies have compared the therapeutic benefits of allogeneic and autologous cells with identical qualities, the immune response against MSCs could be one of the reasons for the lower therapeutic efficacy of MSCs when used in allogeneic situations. Indeed, for the clinical use of MSCs, allogeneic ready-to-use stocks of MSCs will be suitable. The isolation, expansion and validation of the cells is extremely time-consuming, restricting immediate treatment with Good Manufacturing Practices (GMP)-qualified cells at the time of injury or diagnosis. Moreover, several studies have demonstrated inter-individual variabilities conditioned by the quality and effectiveness of each patient’s cells (depending on age, potential disease or treatments). The allogeneic model will guarantee that the cells to be used will be appropriately selected, characterized and stored in line with all GMP requirements. No less importantly, widespread application of the allogeneic model could promote the use of MSC-based therapies, thanks to lower logistics costs compared to those relating to autologous cell procedures.

Thus, in this study, we analyzed *in vivo* the MSC-directed host immune response when embedded in Si-HPMC hydrogel with the aim of enhancing MSC use in clinical practice. The protective effect of the biomaterial against the MSC-host-specific immune response and the pro-regenerative effect of macrophages in the presence of MSCs embedded in hydrogel have been addressed. It is now recognized that the role of the immune system is fundamental in orchestrating the repair response [19]. Biomaterials, depending on their composition (elasticity, porosity, viscosity, etc.) influence biocompatibility with the host organism and the immune reaction. For many years, biomaterials have needed to elicit minimal inflammatory response as that was considered to be an adverse reaction. The paradigm of the host–biomaterial immune response has evolved and been refined, leading to the development of biomaterials that modulate the immune system toward the healing process [20–22]. Thus, we used a xenogeneic transplant model to generate host immune response and develop a set of high-stringency strategies to test the immunomodulatory effects of MSCs associated with Si-HPMC hydrogel. This procedure, designed for studying cellular and antibody responses, will provide valuable information about the action mechanisms of therapeutic effects required for subsequent clinical development of allogeneic MSC therapies combined with biomaterials.

## Methods

### Ethics statement and animals

All experiments were performed in accordance with the Guide for the Care and Use of Laboratory Animals and the French regulations for animal experimentation (French Ministry of Agriculture Order No. B92-032-01, 2006), together with the relevant European Directives (86/609/CEE) and French Decree 2013-118. IRSN (French Institute for Radiological Protection and Nuclear Safety) animal facilities are registered and approved under No. C92-032-01. The experimental protocol was reviewed by IRSN’s Ethics Committee and registered with the CNREEA (*Comité national de réflexion éthique sur l’expérimentation animale,* the French National Committee for the consideration of ethics in animal experimentation) under No. 81. The experimental protocol (P18-03) was submitted to the French national authorization platform and after approval was registered under Permit Number APAFlS#14843-2018042411554405v2. Male Sprague Dawley (non-consanguineous) rats (Janvier SA, France) weighing 300 g were received and housed in a temperature-controlled room (21°C± 1°C). Rats were housed four to a cage and cages have two levels and a red tunnel for shelter. They were allowed free access to water and fed standard pellets (LASQCdiet^®^ Rod16-R, LASvendi, Germany). Rats were anesthetized using isoflurane inhalation (5% flow rate for induction, then 2.5% for anesthesia) and a single 29 Gray (Gy) dose was delivered using the Elekta Synergy Platform, an accelerator-type radiation source (maximum energy is 4 MV with average energy of about 1.5 MV; 30 kA; the dose rate was 2.3 Gy per min) (Elekta SAS France, France) through a 2 × 3 cm window centered on the colorectal region.

### Hydrogel preparation and cell encapsulation

Hydroxypropyl methyl cellulose (Methocel^®^ E4M Premium procured from Dow Chemical) was silanized by grafting 3-glycidoxypropyltrimethoxysilane as previously described [23]. Si-HPMC powder was dissolved in NaOH solution (0.2 M) then dialyzed to reach a final pH value of 12.7. Acidic buffer solution used to neutralize the basic Si-HPMC solution was prepared using 0.1 M HCl and 4-(2-hydroxyethyl)-1-piperazineethanesulfonic acid as previously described [24]. Si-HPMC hydrogels were prepared by rapidly mixing the polymer and the buffer in a ratio of 2:1 to give a pH of 7.2 and then initiating silanol condensation. The injectability of the 1.5% Si-HPMC hydrogel and rheological characteristics were measured as previously described [16] and are given in Table 1.

**Table 1.** Rheological characteristics of the 1.5%Si-HPMC hydrogel

% Si-HPMC	Viscosity (Pas)	Injectability (Kg)	Gel point (min)		Elastic modulus (Pa)	
			Room temperature	37°C	Room temperature	37°C
1.5 %	0.309 ± 0.05	3.52 ± 0.1	41.5 ± 5.9	11.9 ± 1.5	426.8 ± 53.4	380 ± 58.2

For cell encapsulation in the hydrogel, cell pellets were mixed with hydrogel using syringes and a Luer-lock system. Briefly, hydrogel is placed in one syringe and cells in the other, after fixing the two syringes with the Luer lock, and transferring 10 times back-and-forth to allow cell embedding.

### *In vivo* experimental procedure

Three weeks after irradiation, rats were injected with 1.10^6^ of human-MSCs (hu-MSC) from adipose tissue in the submucosa of the colon, using a 29 Gauge needle. Two injection points (2 × 100µl) were used, upstream and downstream of the lesion. After defrosting and 10 days’ culture in a MEMα medium containing 10UI heparin and 0.5% ciprofloxacin with 5% platelet lysate (LP100-Macopharma^®^, France), the hu-MSCs were washed twice in phosphate-buffered saline (PBS), trypsinized and counted, and 1 million cells were embedded or not with Si-HPMC at 1.5%. Animals were sacrificed 1 week later (i.e. 4 weeks following irradiation). Animals were anesthetized by isoflurane inhalation (flow rate 5%), blood was taken by cardiac puncture and mesenteric lymph nodes (MLNs) and colon were collected. Following blood centrifugation (1000 g, 30 min), plasma was collected and stored at –80°C. Lymphocytes were extracted from MLNs using a potter in MEMα medium 0.01% DNase. Total lymphocytes were then frozen at –150°C in dimethyl sulfoxide 10% fetal bovine serum (FBS) for later use. For histological analysis, colons were fixed in 4% formaldehyde and embedded in paraffin.

### Immunohistochemistry

Paraffin-embedded colons were cut on a rotary microtome (Leica Microsystems SAS, France) into serial circular sections of 5 µm, spaced by 150 µm (entire damaged zone). For CD68 (macrophages) immunohistochemistry, sections were dewaxed and then treated with proteinase K (DakoCytomation, France) at room temperature (RT) for 5 min, and quenched for endogenous peroxidases by incubation with 3% H_2_O_2_ in methanol at RT for 10 min. After saturation, mouse anti-rat CD68 1/200 (AbDserotec, UK) was applied to the section for 1 h at 37°C. Polymer anti-mouse horse radish peroxidase (HRP) (GBI Labs, USA) was then incubated for 30 min at RT. For myeloperoxydase (MPO) immunohistochemistry, slides were placed in an antigen retrieval solution (0.01M citrate buffer, pH = 6 (DakoCytomation, France) for 3 × 5 min at 350 W), and after endogenous peroxidases inhibition, Rabbit anti-rat MPO antibody 1/75 (Imgenex, India) was applied to the section for 1 h at 37°C. Polymer anti-rabbit HRP (K4002-DakoCytomation, France) was then incubated for 30 min at RT. Staining was developed using Histogreen substrate (E109; Eurobio, France) and sections were counterstained with Nuclear Fast Red (S1963; DakoCytomation, France), dehydrated and mounted. Isotype control antibodies were used as negative controls. The surface of positive staining (blue pixels) was analyzed on the defined surface using a Leica microscope (Leica Microsystems SAS, France) and Histolab software (MicroVision, USA) and expressed as µm2.

### Detection of MSC-directed antibodies

Antibodies directed against human-MSCs were detected in rat plasma using flow cytometry. One million hu-MSCs were fixed in 4% formaldehyde, then permeabilized using 0.1% Triton-X100. Cells were incubated with 100 µl of plasma from different groups of rats for 1 h at 4°C. Secondary fluorescent Alexa 568 goat anti-rat (A11077-Life Technologies, France) at 1/200 dilution was applied for 30 min at 4°C. After two PBS washing sequences, labeling was analyzed using FACS Canto II and DIVA software (BD Biosciences, France). The results are expressed as median fluorescence intensity (MFI).

### Measurement of lymphocyte-induced MSC apoptosis

Human-MSCs (0.5 million) were incubated with 1.5 µM Cytolight rapid Dye (Green Essen #4705, Essen Bioscience, UK) in a 12-well Falcon plate for 24 h at 37°C. Lymphocytes from MLNs taken from different groups of rats were then incubated at a ratio of 1:10 in lymphocyte culture medium (RPMI 1640, 10% FBS, 50 µM beta mercaptoethanol, 5 µg/ml anti-CD3 Ab and 1 µg/ml anti-CD28 Ab). Annexin V (1/250 annexin V reagent, Essen Bioscience, UK) was added to the medium and the plates were incubated for 76 h in an IncuCyte^®^ (Essen Biosciences, UK) imaging system. Nine pictures per well were taken every hour using a 10× lens and with the live cell imaging system. Double-stained cells were quantified using the IncuCyte^®^ S3 system.

### Macrophage differentiation, co-culture and gene expression analysis

Rat monocytes were isolated and purified from peripheral blood mononuclear cells (PBMCs) of buffy coats using Ficoll-Histopaque 1083 (Sigma, France) in accordance with the manufacturer’s instructions. After isolation from the PBMCs using a CD11b/c selection kit (Miltenyi Biotech, Germany), monocytes were cultured at a density of 0.52 × 10^6^ cells/well in a 24-well plate. Non-adhering cells were removed with washes using a PBS with no Ca2+ or Mg2+. Monocyte-to-macrophage differentiation was induced for 7 days in RPMI 1640, supplemented with 10% FBS, 100 U/ml penicillin, 100 µg/ml streptomycin, 2 mM L-glutamine (all reagents from Gibco, UK) and 100 ng/ml macrophage colony-stimulating factor (Peprotech, France). For macrophage activation, M1 macrophages were stimulated with 50 ng/ml lipopolysaccharides (InvivoGen, UK) and 10 ng/ml Interferon gamma (IFNγ) (Bio-Techne, USA) for 24 h (M1), whereas M2 macrophages were stimulated with 20 ng/ml Interleukin-4 (IL-4) (Bio-Techne, USA) for 24 h (M2) or were untreated for the duration of the culture (M0). Co-culture experiments were performed using transwells on a 24-well plate containing macrophages. Si-HPMC, Hu-MSCs and Hu-MSCs encapsulated in Si-HPMC were incubated in the upper chamber for 24 h. Si-HPMC synthesis and Hu-MSC embedding in Si-HPMC were performed as previously described (Moussa et al.). Following co-culture, macrophages were lysed directly in the culture plate using RNeasy Lysis Buffer (RLT). Total RNA was prepared using a RNeasy Mini Kit (Qiagen, France) and cDNA was obtained using the High Capacity Reverse Transcriptase cDNA Kit (Applied Biosystems, USA). Real-time quantitative polymerase chain reaction (PCR) was analyzed using TaqMan gene expression assay (Applied Biosystems, USA). References for primers and probes are given in Table 2.

**Table 2.** Gene and Taqman gene expression assay reference

Gene symbol	Gene name	Assay ID
cxcl2	C-X-C motif chemokine ligand 2	Rn00586403_m1
il10	Interleukin 10	Rn01483988_g1
il1b	Interleukin 1 beta	Rn00580432_m1
il6	Interleukin 6	Rn01410330_m1
mrc1	Mannose receptor C-Type 1	Rn01483988_g1
nos2	Nitric oxide synthase 2	Rn00561646_m1
Pdgfa	Platelet-derived growth factor Subunit A	Rn00709363_m1
ptgs2	Prostaglandin-endoperoxide synthase 2	Rn01483828_m1
Vegfa	Vascular endothelial growth factor A	Rn01511602_m1
wnt6	Wnt family member 6	Rn00437351_m1
wnt9a	Wnt family member 9A	Rn01496604_m1

The samples (*n* = 6 for each group) were loaded in duplicate and fold changes were calculated using 2^–^^ΔΔCt^ normalizing to 18S, the housekeeping gene.

### Statistical analyses

All data are presented as means ±SEM. Statistical analyses were performed using Graph Pad Prism 8.0 (GraphPad, USA) using raw data. When the group samples passed the Shapiro/Kolmogorov normality tests, statistical analysis was performed by ordinary one-way ANOVA (Tukey’s multiple comparison) with a level of significance of *P* < 0.05. Where they failed the normality tests, the non-parametric Kruskal–Wallis test was used. *P = 0.05; **P < 0.05; ***P = 0.001; ****P < 0.001.

## Results

### Si-HPMC embedding reduces MSC-specific humoral response

**Figure 1.**
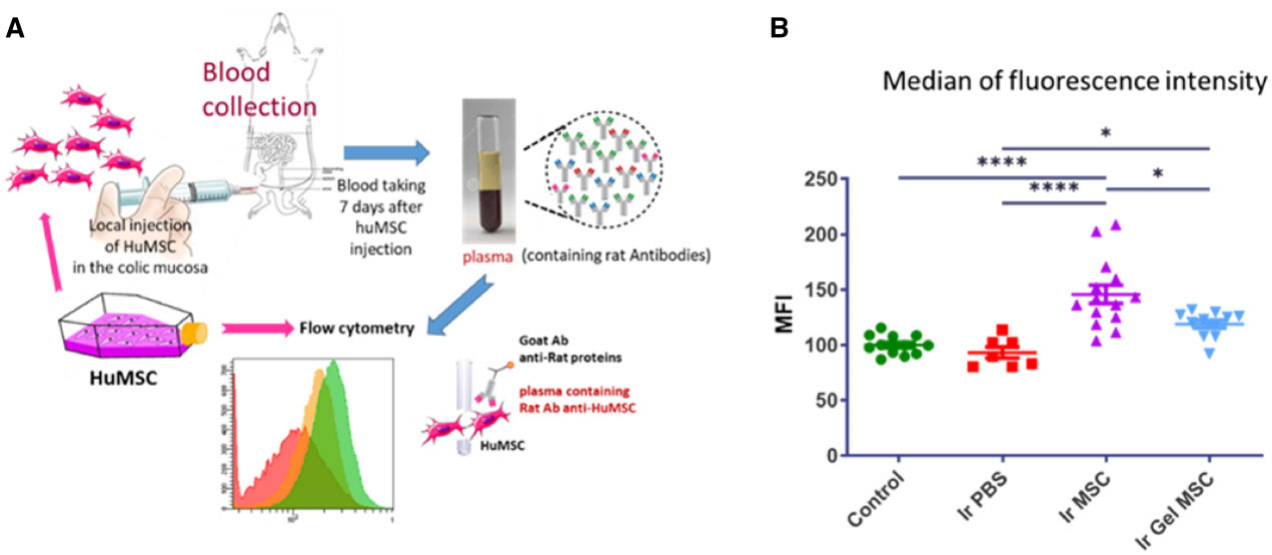
(**A**) Schematic diagram of the procedure to detect plasmatic specific antibodies directed against injected hu-MSCs using flow cytometry. The same hu-MSC batch was used for local *in vivo* injection and labeling and then flow cytometry analysis. (**B**) Scatter plot representing the relative quantity of antibodies in plasma detected in each group of rats by measuring the median fluorescence intensity detected using flow cytometry following permeabilization of the cells. Control = non-irradiated non-injected rats (*n* = 12 animals per group); Ir PBS = irradiated and PBS 1×-injected rats (*n* = 7); Ir MSC = irradiated and hu-MSC-injected rats (*n* = 14); Ir gel MSC = irradiated and Si-HPMC embedded hu-MSC-injected rats (*n* = 12). Statistic *P = 0.05; ****P < 0.001.

We analyzed the rat humoral response specifically directed against injected hu-MSCs. We developed a test using flow cytometry that allowed us to detect labeled hu-MSCs depending on the quantities of anti-hu-MSC antibodies (Ab) present in the rat plasma (Fig. 1A). We compared the MFI in the different groups of rats. Three weeks after colorectal irradiation, rats were injected with hu-MSCs embedded with or without Si-HPMC hydrogel. Plasmas were prepared from blood samples taken after 7 days. Incubating the plasma (containing rat antibodies) with hu-MSCs, from the same batch as those injected in rats, enabled us to fix specific Ab on the cells. Then, following anti-rat secondary fluorescent Ab incubation, quantifications were performed using flow cytometry (Supplementary Fig. S1A). The results were expressed as MFI arbitrarily set to 100 for control rats that did not receive any hu-MSCs (Fig. 1B).

As expected, the irradiated rats with no hu-MSC injection displayed the same MFI level as the control rats. However, the rats that received hu-MSCs showed an increased MFI level (*P* < 0.0001). When hu-MSCs were embedded in Si-HPMC, MFI detection was lower compared to the groups without hydrogel (*P* < 0.05). However, the signal did not return to the basal level (*P* < 0.05). The same experiment was performed 14 days after hu-MSC injection. The results demonstrated increased MFI levels compared to non-injected rats, i.e. with or without Si-HPMC (Supplementary Fig. S1B).

### Si-HPMC protects MSCs from the lymphocyte cytotoxic response

**Figure 2.**
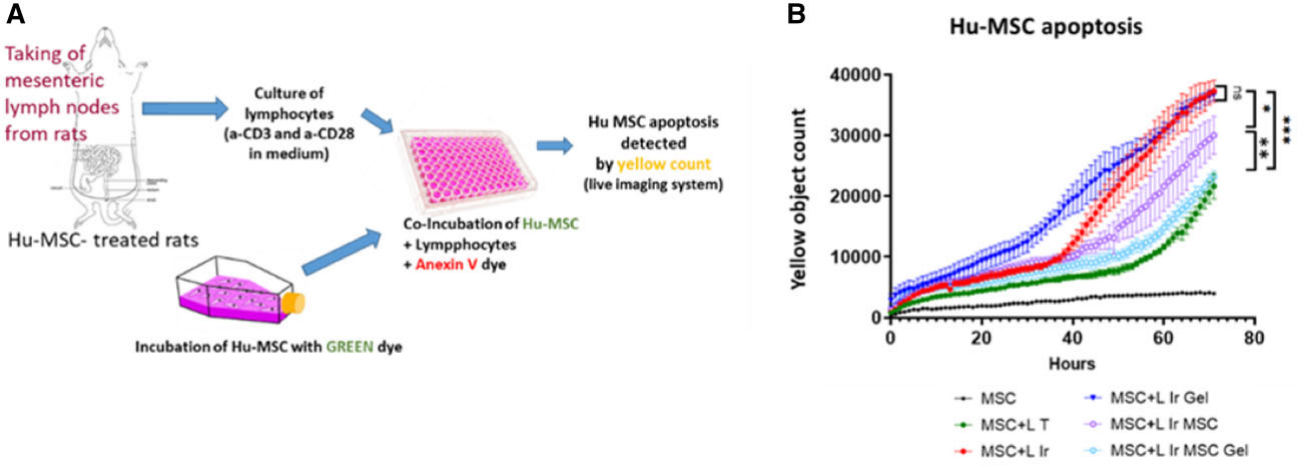
(**A**) Schematic diagram of the procedure used to detect cytotoxic activity against injected hu-MSCs in lymphocytes from the different groups of rats using specific dyes and a live cell imaging system. (**B**) Curves representing the number of apoptotic Hu-MSCs induced after co-incubation with lymphocytes from lymph nodes of the different groups of rats over time. LT = lymphocytes from control rats (*n* = 12 animals per group); L Ir = lymphocytes from irradiated rats (*n* = 7); L Ir gel = lymphocytes from irradiated rats injected with Si-HPMC (*n* = 8); L Ir MSC = lymphocytes from irradiated and hu-MSC-injected rats (*n* = 14); L Ir MSCGel = lymphocytes from irradiated and Si-HPMC embedded hu-MSC-injected rats (*n* = 12). Statistics: *P = 0.05; **P < 0.05; ***P = 0.005.

We investigated the ability of MLN lymphocytes from the different groups of rats to induce apoptosis of hu-MSCs using the live imaging system. We used the same *in vivo* injection protocol and MLN lymphocytes were taken after 7 days. To specifically quantify hu-MSCs that undergo apoptosis, hu-MSCs were first stained with green dye, then co-incubated with lymphocytes from the different groups of rats in the presence of Annexin V (red) dye. Apoptotic hu-MSCs appear as double-stained cells and were quantified over time by live imaging (Fig. 2A).

In our culture conditions, lymphocytes from control rats induced low levels of hu-MSC apoptosis; however, the cytotoxic activity of T cells from irradiated rats was statistically higher (*P* < 0.0001) compared to lymphocytes from non-irradiated animals (Fig. 2B). Using this test, we observed that there was no difference between the cytotoxic activity of lymphocytes from irradiated rats and the cytotoxicity of lymphocytes from irradiated rats injected with hydrogel. Cytotoxic activity of lymphocytes from irradiated rats treated with MSCs was lower than the cytotoxicity of T cells from irradiated rats (*P* = 0.05). Interestingly, the cytotoxicity of lymphocytes from irradiated rats treated with Hu-MSCs embedded in Si-HPMC hydrogel decreased statistically compared to lymphocytes from irradiated rats treated with Hu-MSCs (*P* = 0.001), and was even further reduced compared to irradiated rat injected with Si-HPMC (*P* < 0.001). We observed that treating irradiated rats with Si-HPMC + Hu-MSCs reduced lymphocyte cytotoxicity to reach a similar level of lymphocyte activity as that observed in the control rats (Fig. 2B).

### Macrophage and neutrophil infiltrates after local injection of Si-HMPC in the irradiated colon

**Figure 3.**
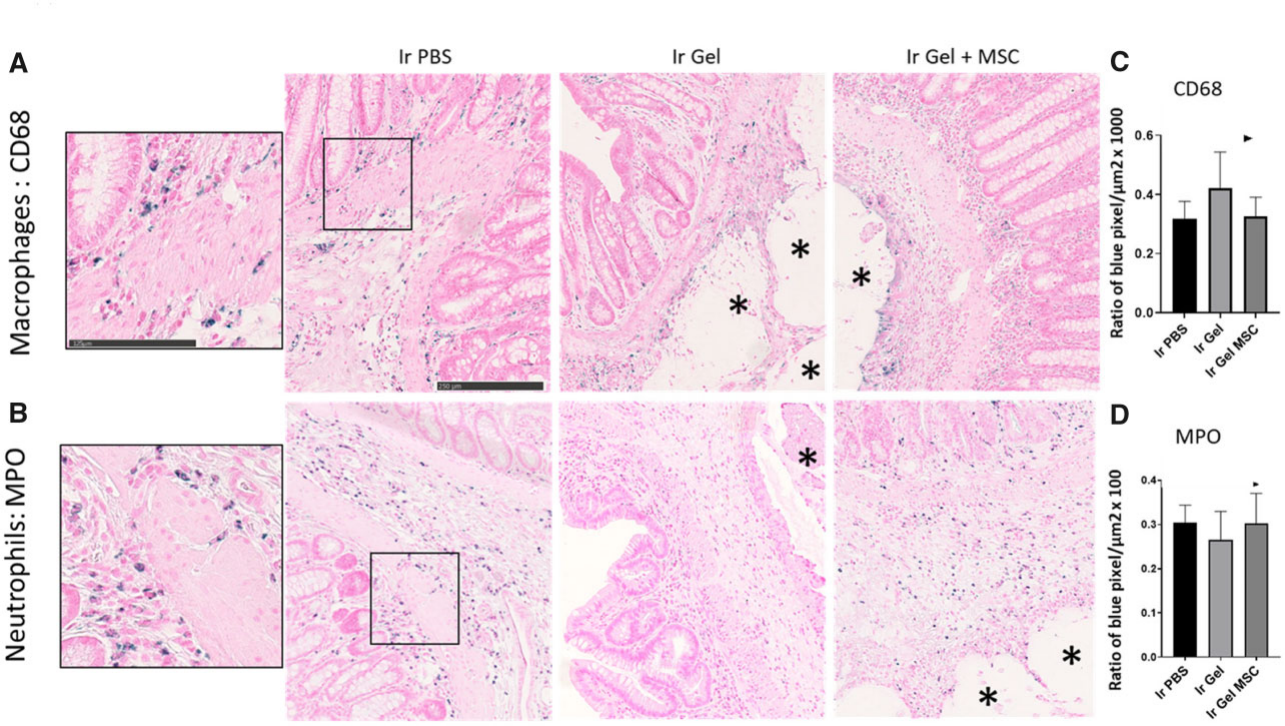
Analysis of immune innate infiltrate in irradiated colonic mucosa using immunohistochemistry. Representative pictures of (**A**) macrophage infiltrate using CD68 immunostaining and (**B**) neutrophil infiltrate using myeloperoxidase (MPO) immunostaining 7 days after *in vivo* injection. Positive signal is colored blue. *Indicates the localization of the gel. (**C** and **D**) Graphs representing the quantification of the blue pixels normalized to a tissue surface located close to the hydrogel and expressed as a percentage. The number of slides analyzed is indicated for each group for CD68 and then MPO immunostaining, respectively. Ir PBS = irradiated and PBS 1×-injected rats (*n* = 16/18); Ir gel = irradiated and Si-HPMC-injected rats (*n* = 10/6); Ir gel MSC = irradiated and Si-HPMC embedded hu-MSC-injected rats (*n* = 16/13).

We used immunohistochemistry to quantify infiltration of innate immune cells in the irradiated colon of rats injected with PBS, Si-HPMC or hu-MSCs embedded in Si-HPMC (Fig. 3). In control rats, few macrophages (CD68 immunostaining) and neutrophils (myeloperoxidase immunostaining) reside in the colonic mucosa (data not shown). After irradiation, some macrophages and neutrophils infiltrated in the mucosa and in the sub-mucosa (Fig. 3A and B, left panels). To study the immune cell infiltrate induced by local injection of Si-HPMC or hu-MSCs embedded in Si-HPMC, the area surrounding the gel was quantified (Fig. 3A and B, middle and right panels). We observed that the presence of Si-HPMC did not increase macrophage and neutrophil infiltrates compared to irradiated colonic mucosa. Quantification did not reveal any statistical differences between the groups (Fig. 3C and D). However, in some animals, we could observe CD68 staining at the periphery of the hydrogel indicating that they could play a role in inducing hydrogel degradation.

### Si-HPMC-embedded Hu-MSCs limit expression of inflammatory molecules by macrophages *in vitro*

**Figure 4.**
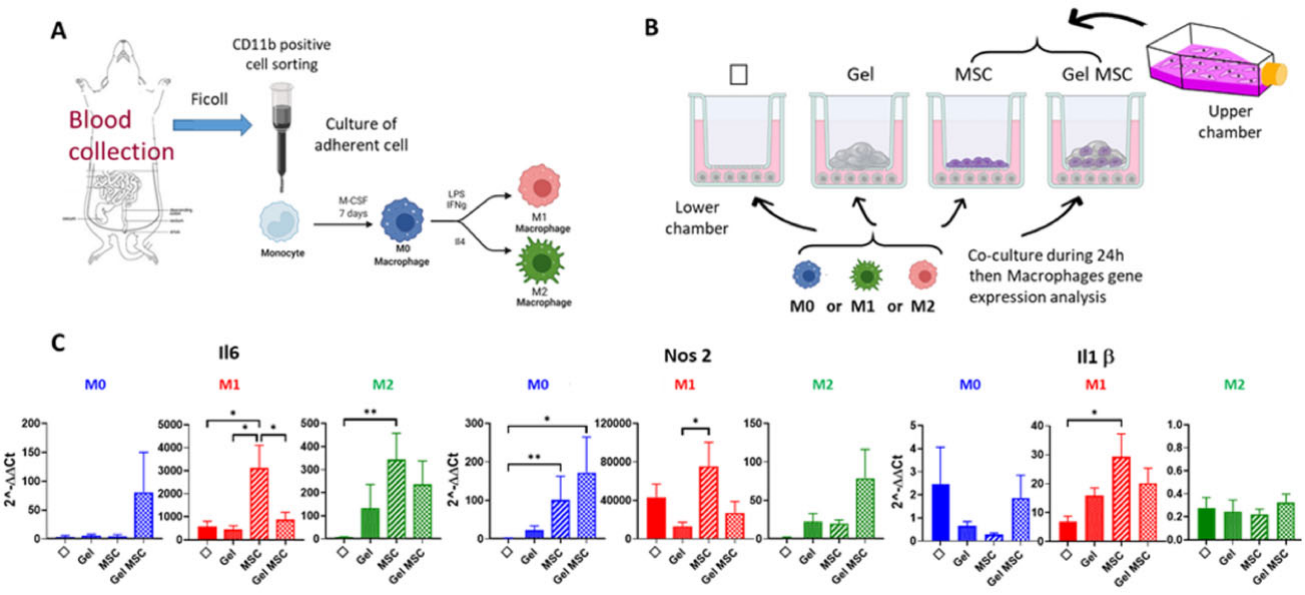
Schematic diagram (**A**) of cell culture method used to obtain macrophages in steady-state (M0), M1 and M2 conditions from the blood of control rats. (**B**) Co-culture method using transwells to evaluate gene expression by macrophages in steady-state (M0), M1 and M2 conditions in the presence of Si-HPMC, Hu-MSCs or Si-HPMC-embedded Hu-MSCs in the upper chamber. (**B**) Histograms represent relative expression by macrophages of genes involved in pro-inflammatory pathways in the different co-culture conditions depending on M0, M1 or M2 stimulation. Results were normalized with 18S housekeeping gene and expressed according to macrophages without co-culture and without stimulation (M0). Experiments were performed three times in triplicate. Φ = macrophages without co-culture; gel = macrophages in the presence of Si-HPMC only in the upper chamber; MSC = macrophage co-culture with hu-MSCs in the upper chamber. Gel MSC = macrophage co-culture with Hu-MSCs embedded in Si-HPMC in the upper chamber. Statistics: *P = 0.05; **P < 0.05.

We further analyzed the interplay between macrophages and Si-HPMC hydrogel embedded or not with Hu-MSCs *in vitro*. Indeed, more and more data have highlighted the importance of pro-regenerative immunity induced by biomaterials in regenerative medicine. Blood monocytes from rats were differentiated *in vitro* in macrophages (Fig. 4A) and then co-cultured using transwells containing Si-HPMC, Hu-MSCs and Hu-MSCs encapsulated in Si-HPMC. Different stimulation conditions have been applied to mimic acute and pro-inflammatory signals (M1) and late and anti-inflammatory signals (M2). No stimulation is used as control (M0-steady state) (Fig. 4B).

We analyzed gene expression of molecules involved in pro-inflammatory macrophages: IL6, NOS2 and IL1β. As expected, they are highly expressed in M1 conditions (×500; ×4000; ×7 respectively, compared to M0). In this M1 condition, Si-HPMC co-culture did not further increase IL6 gene expression by macrophages, unlike hu-MSCs which induced considerable IL6 gene expression (×5; Fig. 4C). Interestingly, encapsulation of hu-MSCs in Si-HPMC limits IL6 increase. NOS2 showed the same expression profile. This limitation effect induced after Hu-MSC encapsulation is also observed to a lesser extent for IL1β. We observed that, in a steady-state situation (M0), Hu-MSCs encapsulated in Si-HPMC increased IL6 and NOS2 (×70; ×150) pro-inflammatory gene expression by macrophages compared to macrophages alone (Fig. 4C, blue panels).

**Figure 5.**
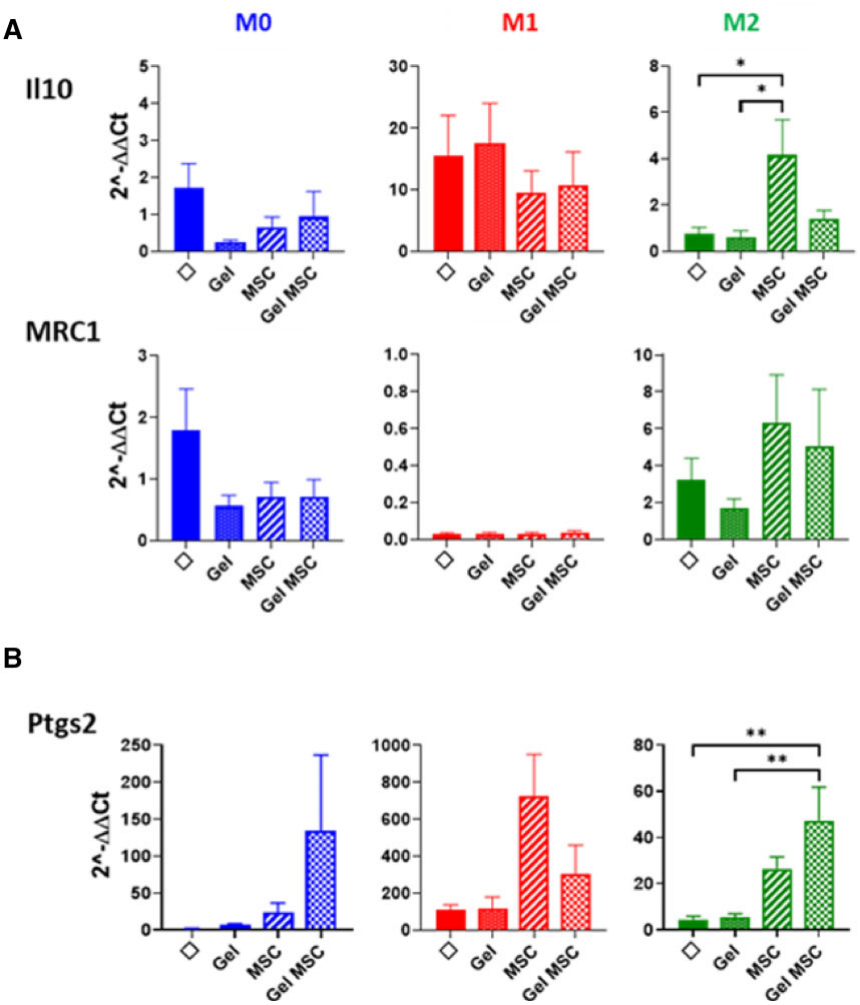
Histograms represent relative expression of genes involved in (**A**) anti-inflammatory pathways and (**B**) gene expression of PTGS2 enzyme by macrophages in the different co-culture conditions according to M0, M1 or M2 stimulation. Results were normalized with 18S housekeeping gene and expressed according to macrophages without co-culture and without stimulation (M0). Experiments were performed three times in triplicate. Φ = macrophages without co-culture; gel = macrophages in the presence of Si-HPMC only in the upper chamber; MSC = macrophage co-culture with hu-MSCs in the upper chamber; Gel MSC = macrophage co-culture with Hu-MSCs embedded in Si-HPMC in the upper chamber. Statistics: *P = 0.05; **P < 0.05.

MRC1 and IL10 has been described in M2 macrophages. MRC1 is expressed by macrophages upon M2 stimulation (Fig. 5A). IL10 expression was observed in M1 conditions, as seen in other studies. Hu-MSCs induced stimulation of IL10 and MRC1 expression by M2-stimulated macrophages. Unlike the MRC gene, Si-HPMC-encapsulated hu-MSC co-culture decreased IL10 expression by macrophages compared to co-culture with non-encapsulated hu-MSC. In steady-state conditions, macrophages did not express IL10 nor MRC1, whatever the co-culture group.

We analyzed PTGS2 enzyme converting arachidonic acid in PGE2, which participates in the pro-inflammatory process and in T-Reg activity. Hu-MSCs co-cultured with or without Si-HPMC, regardless of the stimulation conditions, increased the expression of PTGS2 by macrophages (Fig. 5B). However, after M1 stimulation inducing the highest PTGS2 expression, the encapsulation in Si-HPMC reduces PTGS2 expression. In M0 and M2 conditions, PTGS2 expression by hu-MSCs is not reduced after encapsulation in the hydrogel.

These results demonstrated that macrophages co-cultured with Si-HPMC, Hu-MSCs or Hu-MSCs encapsulated in Si-HPMC induced different outcomes according to steady-state or inflammatory conditions. Hu-MSCs encapsulated in Si-HPMC limit inflammation in inflammatory contexts. However, in a steady-state microenvironment, we observed an increase in inflammatory gene expression by macrophages when they are co-cultured with Hu-MSCs encapsulated in Si-HPMC.

### Si-HPMC- and Si-HPMC-embedded Hu-MSCs induced pro-regenerative and chemo-attractive molecule expression by macrophages *in vitro*

**Figure 6.**
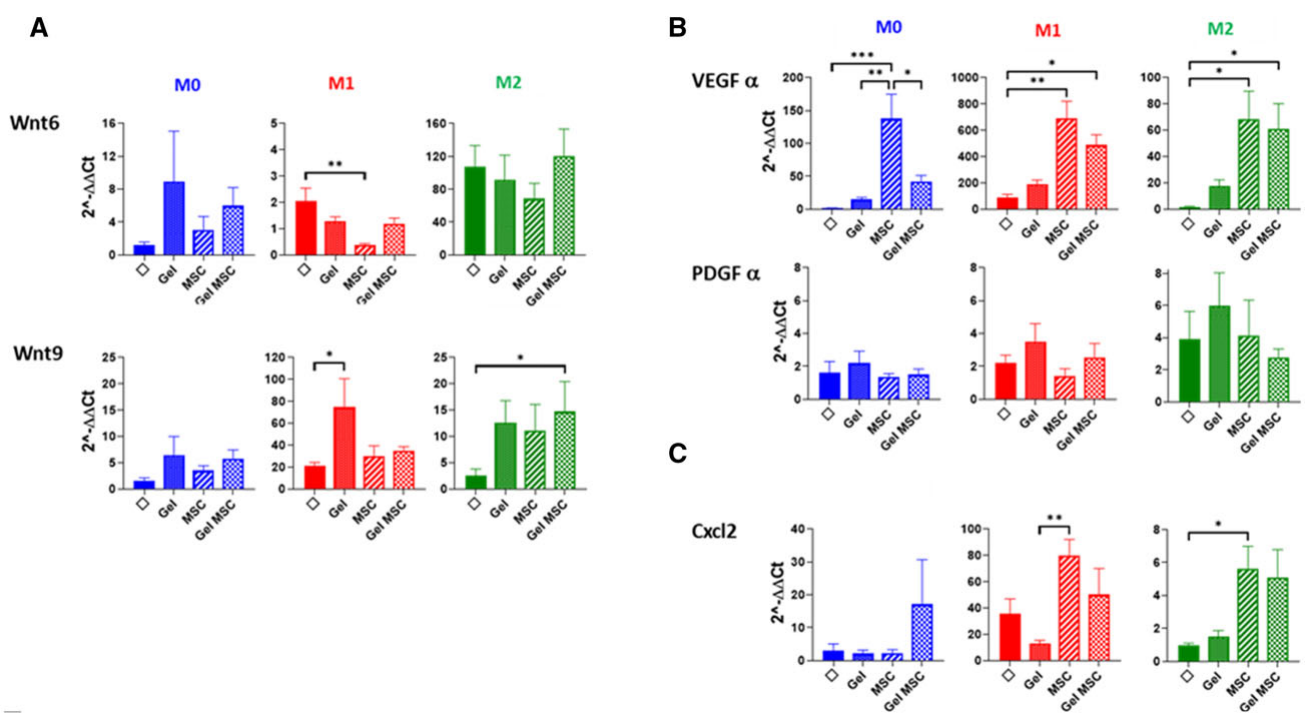
Histograms represent relative expression of genes involved in (**A**) pro-regenerative epithelial process, (**B**) pro-angiogenic process and (**C**) chemoattractant by macrophages in the different groups according to M0, M1 or M2 stimulation in culture. Results were normalized with 18S housekeeping gene and expressed according to macrophages without co-culture and without stimulation. Tm0/Tm1/Tm2 = M0/M1/M2 macrophages from control rats without co-culture (M0). Experiments were performed three times in triplicate. Φ = macrophages without co-culture; gel = macrophages in the presence of Si-HPMC only in the upper chamber; MSC = macrophage co-culture with hu-MSCs in the upper chamber; Gel MSC = macrophage co-culture with Hu-MSCs embedded in Si-HPMC in the upper chamber.

We analyzed gene expression by macrophages of the Wnt family genes and growth factors involved in epithelial proliferation in the colon. Wnt3, Wnt11 and R-spondin were not detected in macrophages in the different culture conditions. EGF (Epithelial growth factor) showed weak detection in macrophages regardless of the culture conditions. IGF (Insulin growth factor) was expressed by macrophages in M0 and M2 but repressed in M1 conditions and few modifications were noticed under the different co-culture conditions (data not shown). Wnt6 was highly induced in macrophages after M2 stimulation (around 100-fold in each culture condition; *P* = 0.003) but not modified between the different groups (Fig. 6A). In M0 conditions, Wnt6 expression by macrophages increased regardless of the co-culture group, compared to macrophages on their own (Fig. 6A). Wnt9 expression increased 2-fold in M1 and M2 conditions compared to M0. Regardless of the microenvironment context, Si-HPMC, Hu-MSCs or Si-HPMC-encapsulated hu-MSCs, we observed an increase in Wnt9 expression by macrophages compared to macrophages on their own (Fig. 6A).

To address the role of macrophages in the vascularization process, we analyzed the expression of VEGFα and PDGFα (Fig. 6B). We observed that Si-HPMC increased expression of VEGFα in macrophages cultured in M0, M1 and M2 conditions, with the highest expression seen under M1 stimulation. Interestingly, we observed that co-culture with Hu-MSCs in M0, M1 and M2 conditions induced considerable VEGFα expression by macrophages (*P* = 0.001, *P* < 0.005 and *P* = 0.05, respectively). We also observed that the encapsulation of hu-MSCs in Si-HPMC increased VEGFα expression in M1- (*P* = 0.05) and M2- (*P* = 0.05) stimulated macrophages. PDGFα expression by macrophages increased in M2 inflammatory conditions. We noticed that the encapsulation of hu-MSCs in Si-HPMC did not modify PDGFα expression by macrophages.

We also analyzed the expression of CXCl2 chemoattractant molecule, involved in cell recruitment. CXCl2 expression in macrophages is high in M1 conditions (Fig. 6C). In M1 and M2 conditions, CXCl2 expression increased when co-cultured with Hu-MSCs and Hu-MSCs encapsulated in Si-HPMC compared to macrophages on their own.

All these data demonstrated that macrophages secrete Wnt6 and VEGFα molecules involved in epithelial regenerative and vascularization processes when they are co-cultivated in transwells with hu-MSCs or Si-HPMC + hu-MSCs. This crosstalk with macrophages could be part of the pro-regenerative action of MSCs, particularly in the case of VEGFα secretion.

## Discussion

This study highlights the potential of MSCs encapsulated in the Si-HPMC hydrogel to modulate the immune and inflammatory responses to support the therapeutic effect [16]. We focused on two aspects; the specific immune response against injected-MSCs and the modulation of innate immune response induced by MSCs encapsulated in a Si-HPMC hydrogel toward a regenerative polarization.

Associating MSCs into biomaterials has been successfully used for bone regeneration, providing 3D organization and supporting cell viability and differentiation ability [25, 26]. Embedding MSCs in hydrogel has already been assessed *in vivo*, albeit to a lesser extent, notably in cell-based myocardium and cartilage regeneration strategies [27–29]. For colonic damage induced by irradiation, we previously tuned the hydrogel and determined that 1.5% Si-HPMC rheological property is compatible with cell embedding and loading through the syringe catheter system of the colonoscope to allow local injection in the colon. Using Green fluorescent protein–mesenchymal stem cells, we demonstrated significant engraftment of cells, which were still alive 7 days after treatment. Some cells were still detected 21 days after local injection in the colon. Using this injection protocol, we found a greater increase in the therapeutic benefits in a rat model compared to intravenous injection of MSCs in saline [16]. Analyses of action mechanisms demonstrated that MSCs encapsulated in Si-HPMC react to a pro-inflammatory environment by secreting pro-angiogenic molecules. Thus, 1.5% Si-HPMC allows the bidirectional diffusion of small molecules (e.g. ions, oxygen, carbon dioxide, growth factors, cellular waste products and therapeutic molecules secreted by the grafted cells) [30]. In this study, we investigated the ability of the hydrogel to isolate the cells from the immune system of the host. Indeed, despite the immuno-privileged status of MSCs, immune rejection has been reported, particularly in re-challenge experiments [31]. Convincing results obtained for an equine model demonstrated that repeated intra-articular injections of allogenic mismatched MSCs increased local inflammation and the development of donor-specific antibodies [32]. Moreover, our team also demonstrated in a mini-pig model that repeated injections of MSCs improve radiation-induced colorectal damage [33]. To provide long-term efficacy of MSCs, iterative injection of allogenic cells is recommended in clinical trials for patients with severe side effects of radiotherapy to the colon (NCT02814864). As allogenic MSCs will be suitable for promoting the clinical use of MSCs, in this study, we analyzed host immune response after the injection of hu-MSCs encapsulated or not in Si-HPMC. Our results demonstrated, in groups of rats irradiated and treated with hu-MSCs without hydrogel, the presence of antibodies and cytotoxic lymphocytes that specifically recognize the injected MSCs. We observed that embedding the cells in the Si-HPMC hydrogel reduced cell-mediated immune response against injected MSCs. Indeed, our tests performed *in vivo* allowed us to detect a reduction in specific antibodies directed against MSCs and a reduction of MSC apoptosis by lymphocytes from MLNs. In the injured brain treated using allogenic MSCs, the presence of cytotoxic CD8 T cells near the transplanted zone has also been observed [34]. To avoid MSC death, cells were embedded in agarose hydrogel which releases Fas ligand to induce apoptosis of surrounding cytotoxic CD8 T cells. Using this strategy, the authors reported increased MSC engraftment, associated with an increase in neurotrophic factors and therapeutic benefit. Taken together, these results suggest that biomaterials used in regenerative medicine have to be optimized to limit host immune response and increase cell engraftment. In the future, testing the therapeutic benefit after re-challenge experiments would be of interest for hydrogel-assisted MSC therapy.

We also determined that the infiltrate of innate immune cells induced after irradiation is not modified by Si-HPMC-encapsulated MSCs compared to non-injected irradiated colonic mucosa, based on immunohistochemistry experiments. Indeed, we observed that the numbers of MPO-positive neutrophils and CD68-positive macrophages near the Si-HPMC are not modified compared to the irradiated mucosa without injection. However, it is well established that macrophage activation and polarization (M1 and M2 phenotypes) control immune response and participate in the tissue healing process. The trend in biomaterial research is to direct the inflammation toward biomaterial integration and tissue regeneration. Moreover, several studies have demonstrated that the therapeutic benefits of intravenously injected MSCs are embolized in the lung and exert their therapeutic benefits by modulating macrophage behavior ([4, 5, 35] and [36] for a review). Interestingly, a recent paper demonstrated that MSC apoptosis and efferocytosis induced immunosuppression of alveolar macrophages and reduced disease severity [5]. We have previously shown that encapsulation of MSCs in hydrogel induces high engraftment of viable MSCs 7 days after local injection in the colon. Hydrogel, mainly composed of water, disappears progressively over time and fewer cells were detected at 14 and 21 days [16]. Thus, we cannot exclude that MSCs are phagocytized by colonic macrophages to exert their immunosuppressive functions. This process could be weak and delayed in time, and thus undetectable using immunohistochemistry (Fig. 3). It is currently technically challenging to sort and analyze macrophages from the colon specifically in proximity to the hydrogel. To mimic the different phases of inflammatory response, we incubated *in vitro* differentiated macrophages in steady-state (M0), M1 (acute response or pro-inflammatory) or M2 (late response or anti-inflammatory) conditions with Hu-MSCs or Hu-MSCs encapsulated in Si-HPMC. First, we observed different macrophage behavior depending on steady-state or inflammatory conditions in response to co-culture with hu-MSCs and MSCs embedded in Si-HPMC. We observed *in vitro* that MSCs significantly increased the quantities of inflammatory genes (IL1β, NOS2 and IL6) secreted by macrophages mainly under inflammatory conditions. This reveals the immunogenic properties of MSCs in non-syngeneic situations. We observed that, in steady-state conditions, embedding hu-MSCs in hydrogel increased the expression of pro-inflammatory genes by macrophages, whereas in M1 conditions, embedding MSCs in Si-HPMC limited the expression of these genes compared to Hu-MSCs alone. This study has highlighted the plasticity of macrophage response depending on co-culture with MSCs or MSCs embedded in hydrogel. Although the importance of macrophage activation is primordial in bone regenerative process *in vivo* [37–39], the effect of M1 or M2 phenotype of macrophages in bone regenerative effect induced by MSCs is controversial [40, 41]. Indeed, it seems crucial in the future that we improve our knowledge on the sequential activation of macrophages during the acute or late phases of the healing process induced by biomaterial-assisted cell therapy. Therefore, given the importance of inflammatory system regulation in the tissue healing process, controlling it through the association of cell therapy and biomaterials is a promising avenue in regenerative medicine.

Cell therapy using MSCs is also based on the ability of MSCs to secrete a wide range of regenerative molecules. It is widely agreed that MSCs are great producers of VEGF, which favors tissue regeneration via the pro-angiogenic process [33, 42, 43]. In this study, we observed that macrophages, co-cultured with hu-MSCs, embedded or not in Si-HPMC, significantly increase VEGF expression. Consequently, our *in vitro* study suggests that some part of VEGF secretion observed in irradiated intestine tissue following MSC treatment could be attributed to macrophage secretion [33, 44]. Wnt family molecules play critical roles in multiple pathways, including stem cell proliferation, self-renewal and tissue regeneration [45, 46]. The importance of Wnt secreted by macrophages has been demonstrated *in vivo* following intestinal injury induced by radiation. Indeed, mice depleted for the porcupine gene, essential for Wnt synthesis, have normal intestinal morphology but are hypersensitive to radiation injury in the intestine [47]. Therefore, therapy that enforces Wnt synthesis would be appropriate to improve intestinal epithelium regeneration. We previously showed that, *in vitro*, MSCs secrete Wnt family members weakly [48]. In this study, we demonstrated that macrophages could be producers of Wnt6 and Wnt9, when they are co-cultivated with Si-HPMC, Hu-MSCs or Si-HPMC-embedded Hu-MSCs, especially in M2 inflammatory conditions. This result highlights a cooperation between the innate immune system and biomaterial-assisted MSC therapy to enhance the epithelium regenerative process.

In conclusion, we have demonstrated, using *in vivo* rat models developing colorectal damage similar to that developed in patients suffering from severe side effects of radiotherapy, that embedding mismatched MSCs in Si-HPMC hydrogel reduced MSC-specific antibodies detected in blood and reduced cell-mediated apoptosis of MSCs by lymphocytes. These results suggest that Si-HPMC protects non-syngeneic MSCs from the host adaptive immune system. Regarding innate immune cells, our study demonstrated that MSCs, whether embedded in Si-HPMC or not, modified VEGF and Wnt gene expression by macrophages, both of which have significant effects on the tissue healing process. Altogether, these data show another aspect in favor of the clinical use of biomaterial-assisted cell therapy integrating the immune system into regenerative strategies, thereby opening up attractive possibilities for the use of universal donors for therapeutic applications.

## Supplementary data

Supplementary data are available at *REGBIO* online.

## Supplementary Material

rbac022_Supplementary_Data
