# Supplementary material for: Embedding MSCs in Si-HPMC hydrogel decreased MSC-directed host immune response and increased the regenerative potential of macrophages
Source: Regen Biomater. 2022 Apr 25;9:rbac022. doi: 10.1093/rb/rbac022 (PMC9245650; doi:10.1093/rb/rbac022)
Supplement: rbac022_Supplementary_Data [file rbac022_supplementary_data.pdf]

Supplementary Data 1. *Schematic diagram of the gating procedure for flow cytometry analyses. Hu-MSC were visualized according to SSC (granularity) and FSC (size) using BD Bioscience flow cytometer. Then, median of Phycoerythrin (PE) fluorescence was quantified on cells in singlet.*

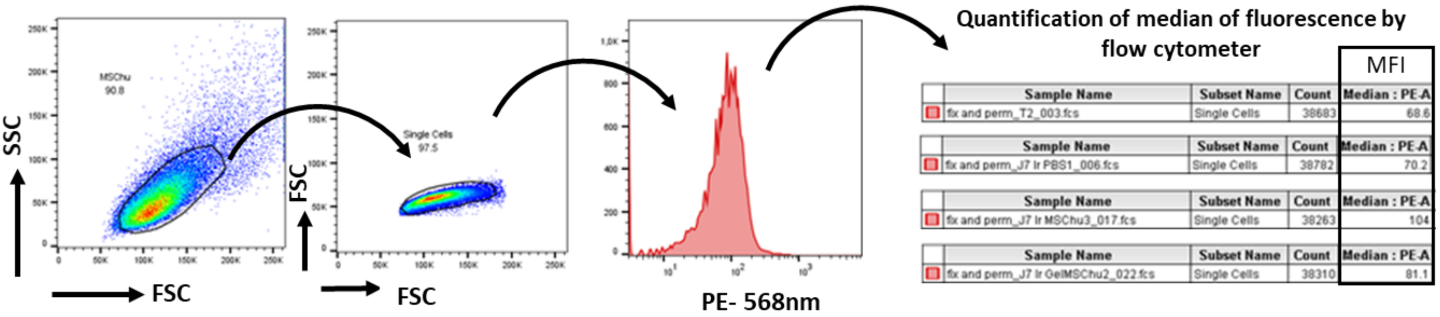

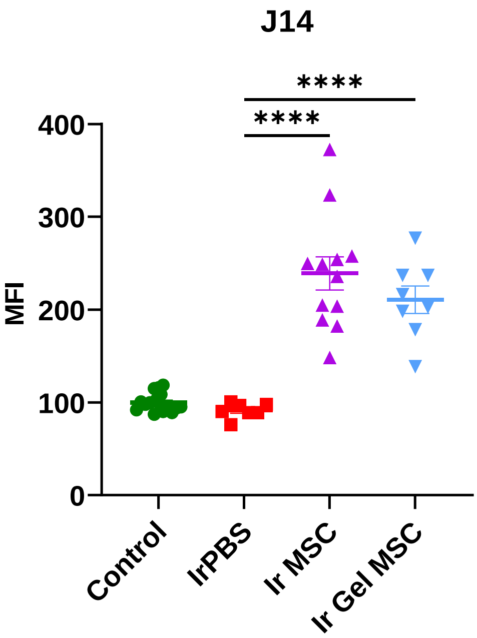

Supplementary Data 2. *Scatter plot representing the relative quantity of antibodies in plasma detected in each group of rats 14 days after injection of hu-MSC by measuring the median fluorescence intensity detected using flow cytometry following permeabilization of the cells. Control = non-irradiated non-injected rats (n=16 animals per group); Ir PBS = irradiated and PBS 1x-injected rats (n=7); Ir MSC = irradiated and hu-MSC-injected rats (n=12); Ir Gel MSC = irradiated and Si-HPMC embedded hu-MSC-injected rats (n=8). Statistic **** p<0.001.*
